# Evaluate the effects of low-intensity pulsed ultrasound on dental implant osseointegration under type II diabetes

**DOI:** 10.3389/fbioe.2024.1356412

**Published:** 2024-02-02

**Authors:** Yingying Wang, Ximeng Cao, Yingyi Shen, Qi Zhong, Ziang Wu, Yaqin Wu, Weimin Weng, Chun Xu

**Affiliations:** ^1^ Department of Prosthodontics, Shanghai Ninth People’s Hospital, Shanghai Jiao Tong University School of Medicine, Shanghai, China; ^2^ College of Stomatology, Shanghai Jiao Tong University, Shanghai, China; ^3^ National Center for Stomatology and National Clinical Research Center for Oral Diseases, Shanghai, China; ^4^ Shanghai Key Laboratory of Stomatology, Shanghai Research Institute of Stomatology, Shanghai, China

**Keywords:** low-intensity pulsed ultrasound, implantation, type II diabetes mellitus, osteogenesis, osseointegration

## Abstract

**Objective:** The objective of this study is to assess the impact of low-intensity pulsed ultrasound (LIPUS) therapy on the peri-implant osteogenesis in a Type II diabetes mellitus (T2DM) rat model.

**Methods:** A total of twenty male Sprague-Dawley (SD) rats were randomly allocated into four groups: Control group, T2DM group, Control-LIPUS group, and T2DM-LIPUS group. Implants were placed at the rats’ bilateral maxillary first molar sites. The LIPUS treatment was carried out on the rats in Control-LIPUS group and T2DM-LIPUS group, immediately after the placement of the implants, over three consecutive weeks. Three weeks after implantation, the rats’ maxillae were extracted for micro-CT, removal torque value (RTV), and histologic analysis.

**Results:** Micro-CT analysis showed that T2DM rats experienced more bone loss around implant cervical margins compared with the non-T2DM rats, while the LIPUS treated T2DM rats showed similar bone heights to the non-T2DM rats. Bone-implant contact ratio (BIC) were lower in T2DM rats but significantly improved in the LIPUS treated T2DM rats. Bone formation parameters including bone volume fraction (BV/TV), trabecular thickness (Tb.Th), bone mineral density (BMD) and RTV were all positively influenced by LIPUS treatment. Histological staining further confirmed LIPUS’s positive effects on peri-implant new bone formation in T2DM rats.

**Conclusion:** As an effective and safe treatment in promoting osteogenesis, LIPUS has a great potential for T2DM patients to attain improved peri-implant osteogenesis. To confirm its clinical efficacy and to explore the underlying mechanism, further prospective cohort studies or randomized controlled trials are needed in the future.

## 1 Introduction

Low-intensity pulsed ultrasound (LIPUS) is a noninvasive therapy that harnesses acoustic pulsed energy to provide physical stimulation. The research on LIPUS has been ongoing for approximately two decades. Ultrasound has been found to promote various biological processes, including the formation of bone matrix by osteoblasts, the synthesis of collagen by fibroblasts, the synthesis of aggrecan in chondrocytes, and the differentiation of bone mesenchymal stem cells (BMSC) into osteoblasts on titanium surfaces (An et al., 2018). LIPUS treatment increased the number of mineralization nodules of microfilaments, pseudopods of the cells and the amount of extracellular matrix (An et al., 2018).

With the development of the materials and technologies, dental implants emerged as a popular solution for restoring missing teeth in patients. Osseointegration, a crucial aspect for successful implant fixation (Chauvel-Picard et al., 2022), was first conceptualized by Brånemark in 1965 (Brånemark et al., 1977). It refers to the direct contact between a biomaterial and bone tissue without the presence of fibrous tissue in between.

Several factors might influence the establishment of osseointegration, one of which is Type II diabetes mellitus (T2DM) condition. Bone re-modelling disorders resulting from diabetes poses significant risks to the overall success of dental implant treatments (Hua et al., 2018; Ding et al., 2019; Meza Maurício et al., 2019). T2DM has a high prevalence especially in the elderly, while among which the need for restoring the missing teeth is high (Petersmann et al., 2018). Studies have showed that T2DM can exert detrimental effects on various organs and tissues (Ren et al., 2019; Guo et al., 2020). Human blood carries a substantial amount of oxygen and active proteins, which can influence the proliferation and differentiation of bone-derived cells surrounding the implant. Previous studies have indicated that patients with diabetes mellitus exhibit diminished proliferation and osteogenic differentiation capabilities of osteoblasts compared to individuals without diabetes (Li et al., 2015; Graves et al., 2020). Furthermore, the presence of elevated blood glucose levels (Li et al., 2015; Przekora et al., 2016) and disruptions in calcium or phosphorus metabolism, commonly observed in individuals with T2DM, can significantly impact bone tissue remodeling and interfere with the bonding between implants and bone, particularly within the first three weeks following implant placement (Wang et al., 2021).

For achieving a high-quality implant fixation, it is essential to have a rapid and successful osseointegration (Ustun et al., 2008; Meng et al., 2016). Various methods have been developed to stimulate osteogenesis. Among these approaches, ultrasounds, specifically LIPUS, have been proven to be effective in stimulating bone, cartilage, tendon and mucosal regeneration (Khanna et al., 2009; Lavandier et al., 2009; Hsu et al., 2011; Padilla et al., 2016; Chauvel-Picard et al., 2021). Multiple studies have investigated the potential benefits of LIPUS in promoting the osseointegration of dental implants using animal models (Park et al., 2007; Suh et al., 2007; Ustun et al., 2008; Mathieu et al., 2011; Liu et al., 2012; Wang et al., 2023a). However, whether LIPUS could promote osteogenesis around dental implant under T2DM condition has not been reported.

Drawing from these studies on the use of LIPUS in facilitating bone regeneration around implants, it is reasonable to speculate that LIPUS could serve as a valuable supplementary treatment approach to enhance implant osseointegration in individuals with diabetes. Therefore, this study evaluated the effects of LIPUS treatment on osteogenesis around dental implant in a T2DM rat model, by which to provide a reference for future studies to achieve better prognosis of implant treatment in T2DM patients.

## 2 Materials and methods

The entire process followed the guidelines specified in the Animal Research: Reporting *In Vivo* Experiments (ARRIVE protocol) (Percie du Sert et al., 2020). Approval for this study was obtained from the Experimental Animal Ethics Committee of the Ninth People’s Hospital, affiliated with Shanghai Jiao Tong University School of Medicine (reference number: SH9H-2022-A84-1).

### 2.1 Animals

Male Sprague-Dawley (SD) rats, aged 7 weeks, were acquired and subsequently housed at room temperature around 20°C with a 12-hour light/dark cycle.

### 2.2 Group assignment

**FIGURE 1:**
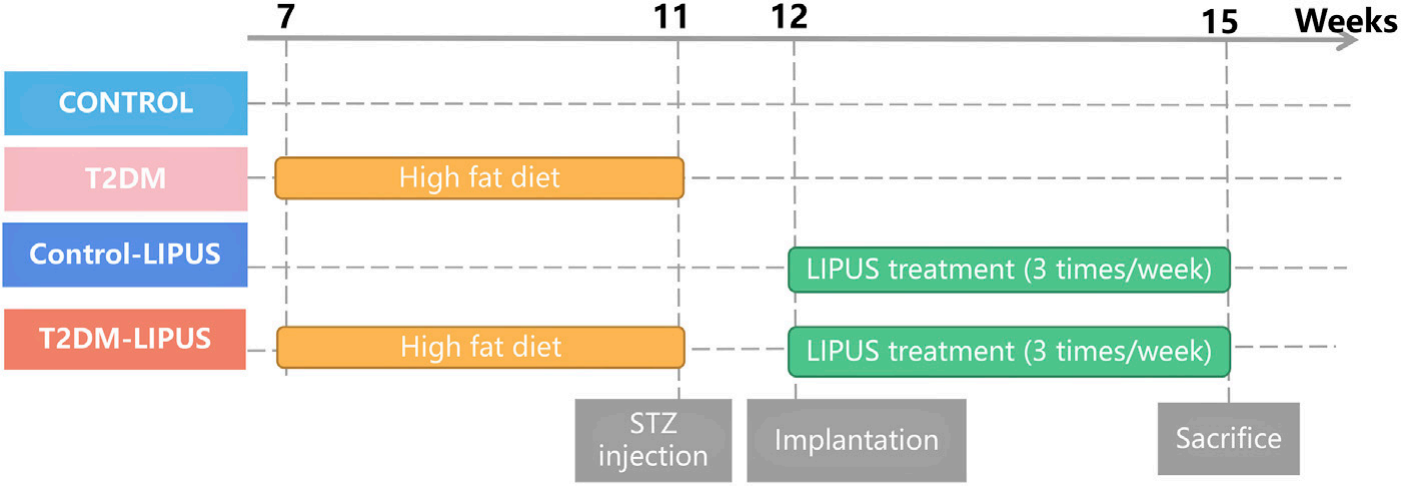
Group assignment. T2DM, type II diabetes mellitus; LIPUS, low-intensity pulsed ultrasound; STZ, streptozotocin.

Following Mead’s resource equation and considering outcome variance and various treatments, the estimated sample size remained consistent with that employed in previous studies (Ganzorig et al., 2015; Ruppert et al., 2019; Sun et al., 2020). The SD rats were randomly divided into 4 groups, 5 rats in each group, as follows: (a) Control group, (b) T2DM group: with experimentally induced T2DM before implantation surgery, (c) Control-LIPUS: after implantation surgery, the implant sites were treated with LIPUS for 3 consecutive weeks, and (d) T2DM-LIPUS group: with experimentally induced T2DM before implantation surgery, and the implant sites were treated with LIPUS for 3 consecutive weeks after implantation surgery. The group assignment and the entire clinical procedure are depicted in Figure 1.

### 2.3 Induction of T2DM

The rats were provided with a high-fat diet for a duration of 4 weeks. Following the high-fat diet regimen, the rats received an intraperitoneal injection of 35 mg/kg streptozotocin (STZ) to induce a state of T2DM (Li et al., 2020; Chen et al., 2021; Khater et al., 2021). Rats with fasting blood glucose (FBG) levels exceeding 16.7 mmol/L 3 days after the STZ injection were considered successfully established T2DM models.

### 2.4 Implantation surgery

**FIGURE 2:**
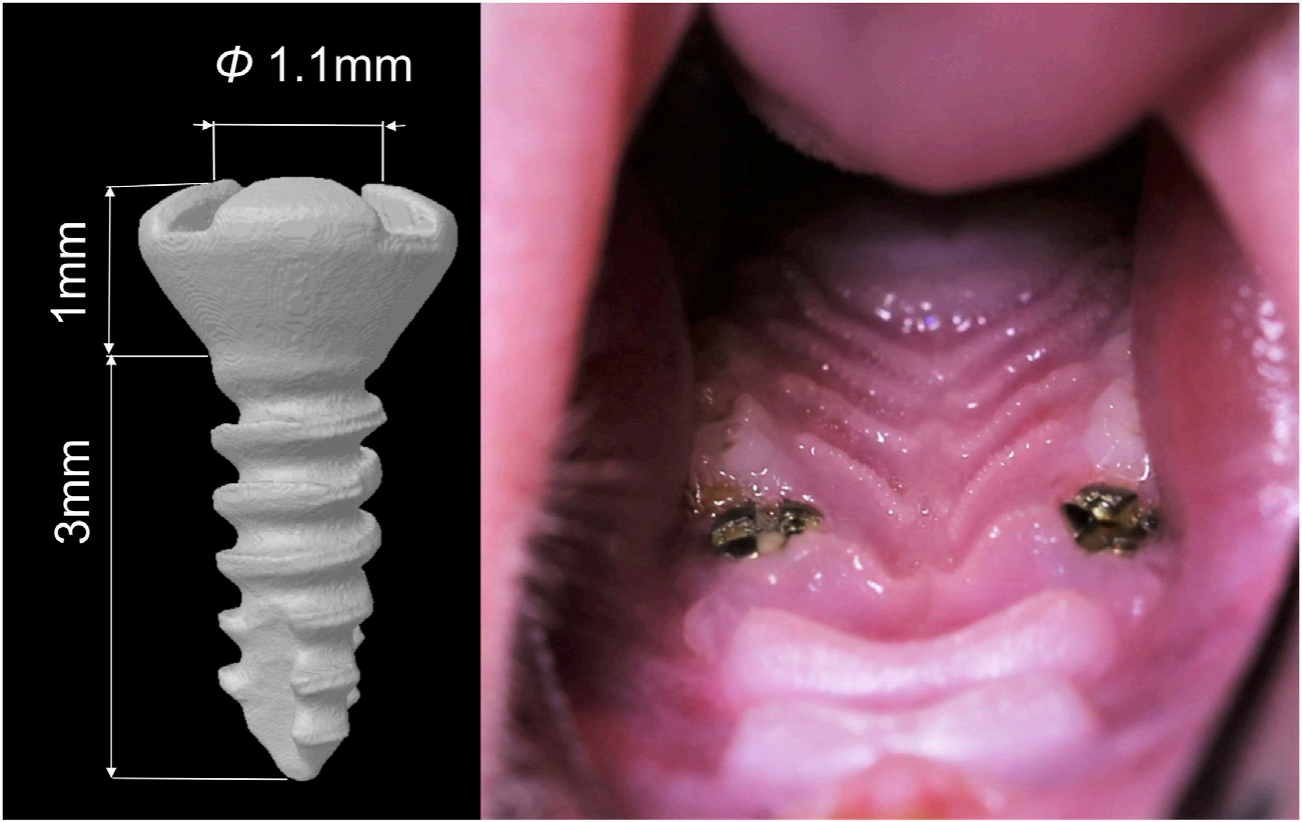
Diagram of the dental implant used in the study and the intra-oral photographs of the rat after implantation surgery.

This implantation surgery in rats maxillae has been reported and evaluated in our previous study (Wang, 2023b), and the procedure was as follows. One week before the surgery, the rats were administered daily antibiotics through oral infusion, with a dosage of 100 μL, including 20 mg of kanamycin and 20 mg of ampicillin. After the rats were anesthetized by administering ketamine (85–90 mg/kg body weight) and xylazine (5–10 mg/kg body weight) intraperitoneally, the bilateral maxillary first molars were extracted. Sockets were rinsed with saline. After socket rinsing, a low-speed handpiece was utilized, operating at a drill diameter of 1 mm and a speed of 1,000 rpm, with continuous irrigation of cooled saline solution to prepare the implant sites in the palatal root socket area of the extraction site. Subsequently, a titanium alloy self-tapped implant (Ti-6Al-4V with an anodized surface, Baiortho^TM^, China) was inserted and allowed to heal transmucosally (Figure 2). Throughout the initial week of the healing period, the rats received a daily antibiotic dose (consistent with the previous dosage). Their oral and overall health was regularly monitored. At the conclusion of the experiment (3 weeks post-implantation surgery), the rats were humanely euthanized using an anesthesia overdose.

### 2.5 Insonification

**FIGURE 3:**
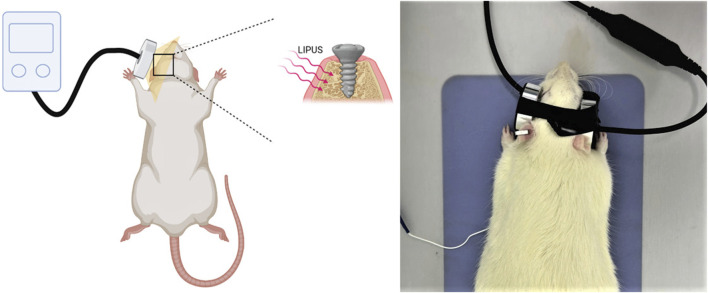
LIPUS treatment. LIPUS, low-intensity pulsed ultrasound.

The rats were anesthetized through intraperitoneal administration of ketamine (85–90 mg/kg body weight) and xylazine (5–10 mg/kg body weight). The implant placement site received LIPUS (Osteotron IV, Ito Co., Ltd, Japan) for a duration of 20 min per day, 3 days per week, over a period of 3 consecutive weeks after the implantation surgery, with a 60 mW/cm^2^ average temporal and spatial intensity and a 1.5 MHz operation frequency (Figure 3). The parameters of LIPUS treatment were taken from our previous study results (Wang et al., 2023a).

### 2.6 Removal torque test

At the conclusion of the experiment (3 weeks post-implantation surgery), the rats’ maxillae were collected, and the Removal Torque Value (RTV) was assessed. RTV was defined as the maximum rotational force applied in the reverse direction until the implant became horizontally rotatable.

### 2.7 Micro-CT analysis

The maxillae of the rats were collected and subjected to scanning using a micro-CT (Skyscan 1076, Belgium) with a resolution of 9 μm. A Volume of Interest (VOI) was the area within a 500 μm radius around the implants, covering the peri-implant region. The 3D reconstructions of the maxillae with the placed dental implants were established from the micro-CT scanning and the marginal bone heights around the implants in the rats of different groups were marked. Subsequently, the bone tissue evaluation script generated the final segmented bone image, encompassing the following parameters: marginal bone loss, trabecular thickness (Tb.Th), trabecular number (Tb.N), trabecular separation (Tb.Sp), bone volume fraction (BV/TV), bone mineral density (BMD), bone-implant contact ratio (BIC), and bone surface density (BS/TV). The measurement method of marginal bone loss is to select the section along the long axis of the implant and also including the central axis of the implant. According to the above section and the implant actual length, the marginal bone loss = (4 - the length of the intraosseous implant/4) mm (Sun et al., 2020).

### 2.8 Histologic processing

Three weeks after surgery, the animals were euthanized with an overdose of anesthesia. The specimens underwent fixation, decalcification, dehydration, and embedding. Subsequently, all specimens were sectioned parallel to the long axis of the implant. The hematoxylin and eosin (H&E) staining and Masson staining were applied to detect the new bone formation around the implant. Images were taken under a microscope (CX33, Olympus, Japan).

### 2.9 Statistical analysis

The data expressed as mean ± standard deviation was analyzed using SPSS version 26.0 (IBM, Chicago, IL, USA). The differences among the three groups were compared using a one-way ANOVA and the following LSD test. The statistical significance was when the *p*-value was less than 0.05.

## 3 Results

### 3.1 Implant survival rate

Three weeks after the implant surgery, the implant survival rate was recorded as showed in Table 1 (implant survival rate = number of final residual implants/number of placed implants). The implant survival rate was 80% in all four groups.

**TABLE 1:** The implant number and implant survival rates of different groups.

	Control	Control-LIPUS	T2DM	T2DM-LIPUS
Number of rats	5	5	5	5
Number of placed implants	10	10	10	10
Number of final residual implants	8	8	8	8
Implant survival rate	80%	80%	80%	80%

LIPUS, low-intensity pulsed ultrasound; T2DM, type II diabetes mellitus.

### 3.2 Evaluation of T2DM rats

**FIGURE 4:**
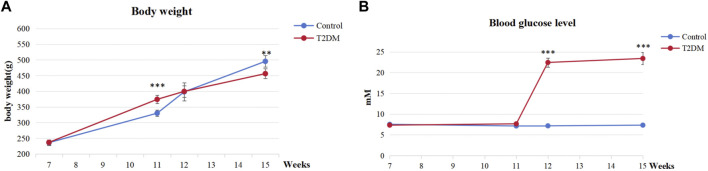
Evaluation of T2DM rats. **(A)** Body weight at various time points. **(B)** Blood glucose levels at various time points. ***p < 0.001, **p < 0.01.

Our findings indicated that all rats induced by a high-fat diet and STZ exhibited T2DM. Illustrated in Figure 4A, the rats in the T2DM group exhibited lower body weight than those in the control group, indicative of typical T2DM symptoms. As presented in Figure 4B, a week post-STZ injection, the T2DM group showed a significantly elevated blood glucose level compared to the control group, maintaining levels more than 16.7 mmol/L throughout the experiment. These results indicated the successful establishment of T2DM rat models with hyperglycemia.

### 3.3 Analysis of the micro-CT

**FIGURE 5:**
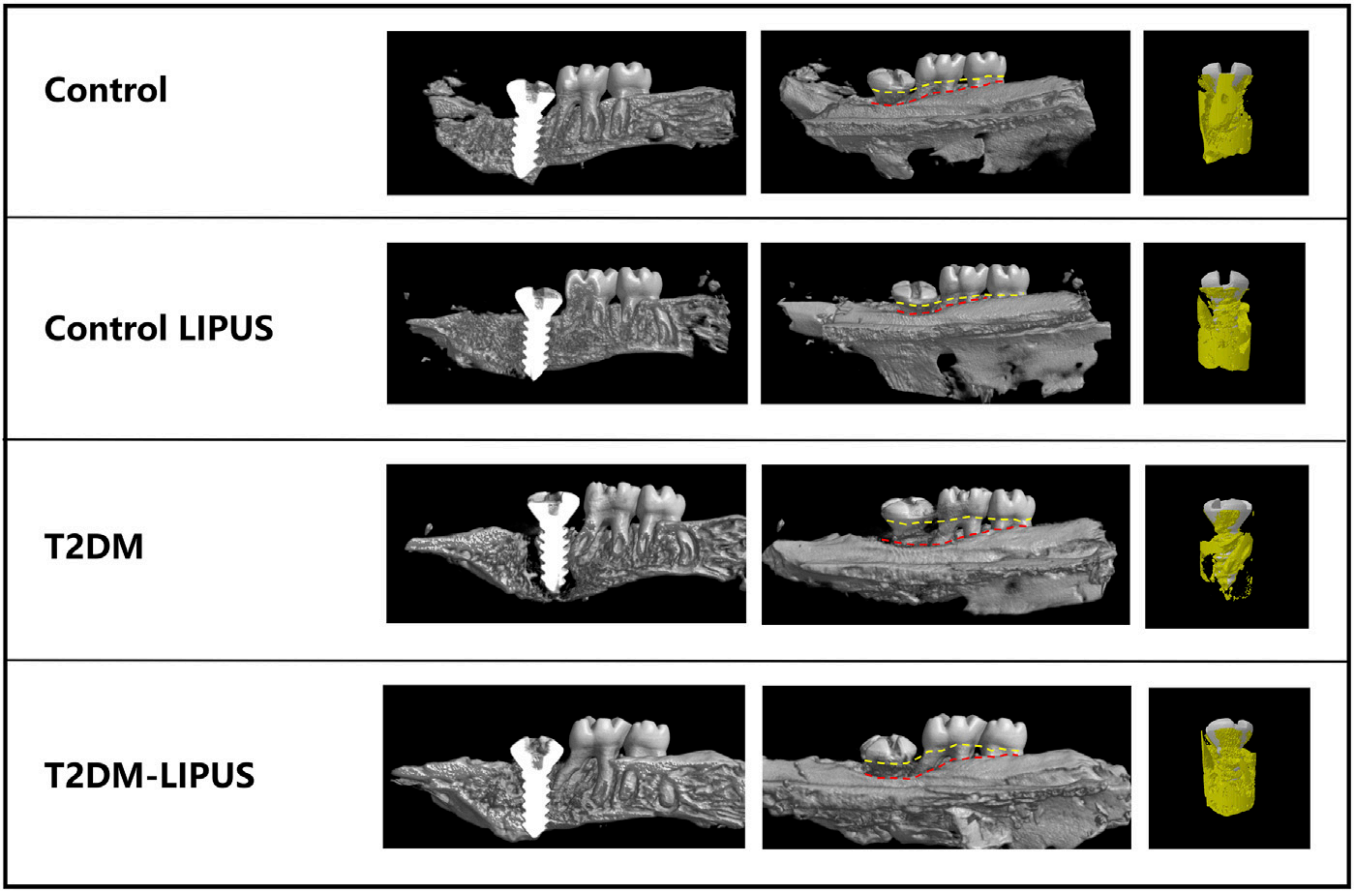
Three-dimensional visualization of the dental implant using Micro-CT technology. The marginal bone loss is assessed by the distance between the yellow and red line in the long axis direction of implants. Yellow line refers to the top of implant and cemento-enamel junction of the second and third molars. Red line refers to the marginal bone level. T2DM group showed significantly more marginal bone loss than other groups. T2DM, type II diabetes mellitus; LIPUS, low-intensity pulsed ultrasound.

**FIGURE 6:**
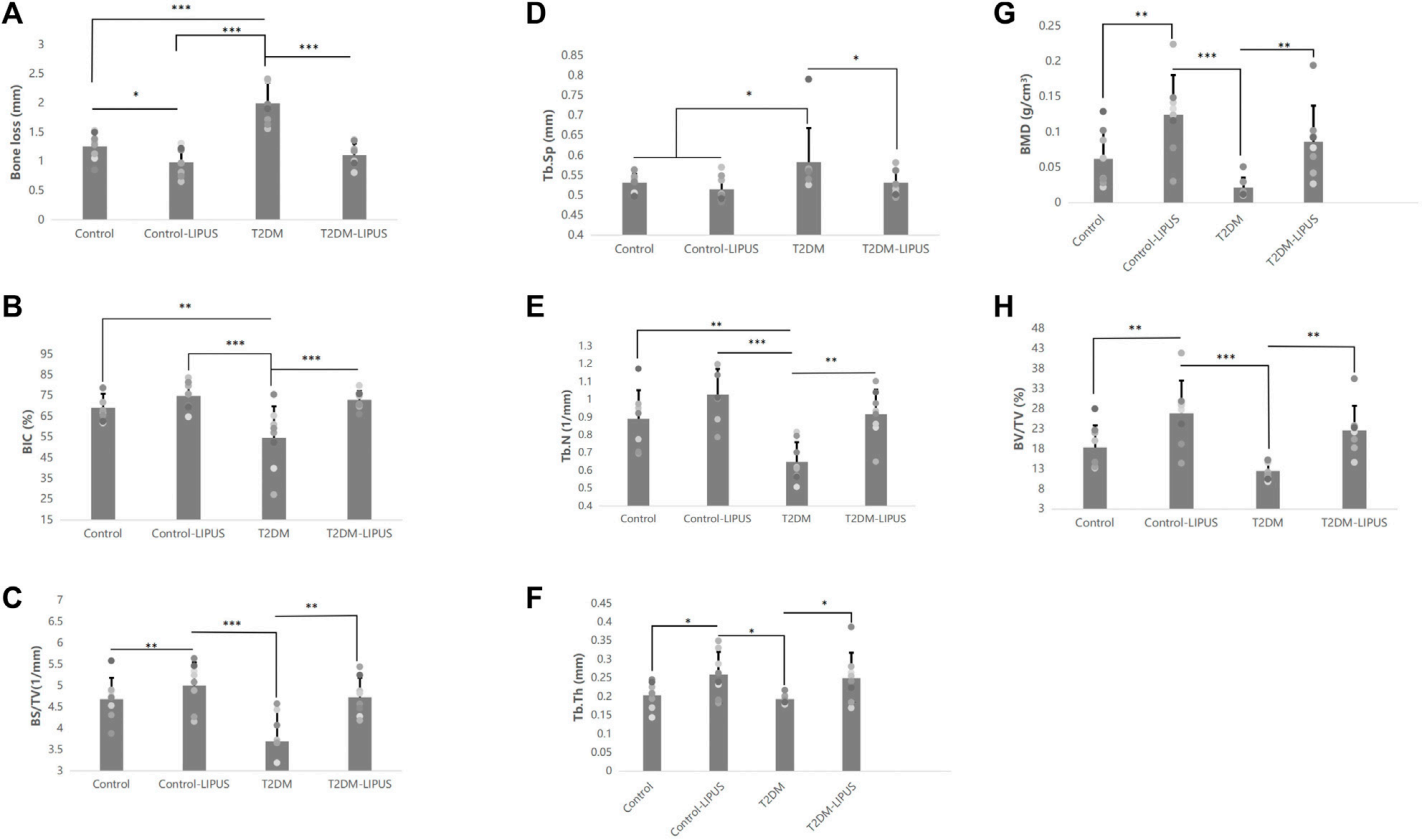
Results of the Micro-CT analysis of marginal bone loss, BIC, BS/TV, Tb.Sp, Tb.N, Tb.Th, BMD, and BV/TV. **(A)** Results of the micro-CT analysis of marginal bone loss; **(B)** Results of the micro-CT analysis of BIC; **(C)** Results of the micro-CT analysis of BS/TV; **(D)** Results of the micro-CT analysis of Tb.Sp; **(E)** Results of the micro-CT analysis of Tb.N; **(F)** Results of the micro-CT analysis of Tb.Th; **(G)** Results of the micro-CT analysis of BMD; **(H)** Results of the micro-CT analysis of BV/TV. The results showed that T2DM group had significant lower BIC ratio and Tb.N than control group, and significant higher Tb.Sp and greater marginal bone loss than control group. LIPUS treatment significantly increased BIC, Tb.N, Tb.Th, BMD, BV/TV and BS/TV, and significantly decreased marginal bone loss and Tb. Sp of T2DM rats. LIPUS treatment also significantly increased Tb.Th, BMD, BV/TV and BS/TV, and significantly decreased marginal bone loss of healthy rats. There was no difference between Control and T2DM-LIPUS groups in all the above indexes. *: *p* < 0.05, **: *p* < 0.01,***:*p* < 0.001. Tb.N, trabecular number; Tb.Th, trabecular thickness; Tb.Sp, trabecular separation; BV/TV, bone volume fraction; BMD, bone mineral density; BIC, bone-implant contact ratio; BS/TV, bone surface density; LIPUS, low-intensity pulsed ultrasound; T2DM, type II diabetes mellitus.

The 3D reconstructions of the maxillae with the placed dental implants were shown in Figure 5, demonstrating the marginal bone height around the implants of the rats in different groups. There was significantly more bone loss at the implant cervical margin area in T2DM groups, but T2DM-LIPUS group showed similar marginal bone height with Control group, as showed in Figure 6A. BIC ratio was presented in Figure 6B, showing that T2DM group had significant lower BIC ratio than Control group. T2DM-LIPUS group had significant higher BIC ratio than T2DM group. BS/TV ratio was presented in Figure 6C, showing that LIPUS treatment increased BS/TV ratio of control and T2DM groups significantly. Tb.Sp was presented in Figure 6D, which showed that T2DM group had significant higher Tb.Sp than other three groups. Tb.N was presented in Figure 6E, which showed that T2DM group had significant lower Tb.N than other three groups. Tb.Th was presented in Figure 6F, which showed that LIPUS treatment increased Tb.Th of control and T2DM groups significantly. BMD was presented in Figure 6G, showing that LIPUS treatment increased BMD of control and T2DM groups significantly. BV/TV ratio was presented in Figure 6H, which showed that LIPUS treatment increased BV/TV ratio of control and T2DM groups significantly.

### 3.4 Analysis of the RTV

**FIGURE 7:**
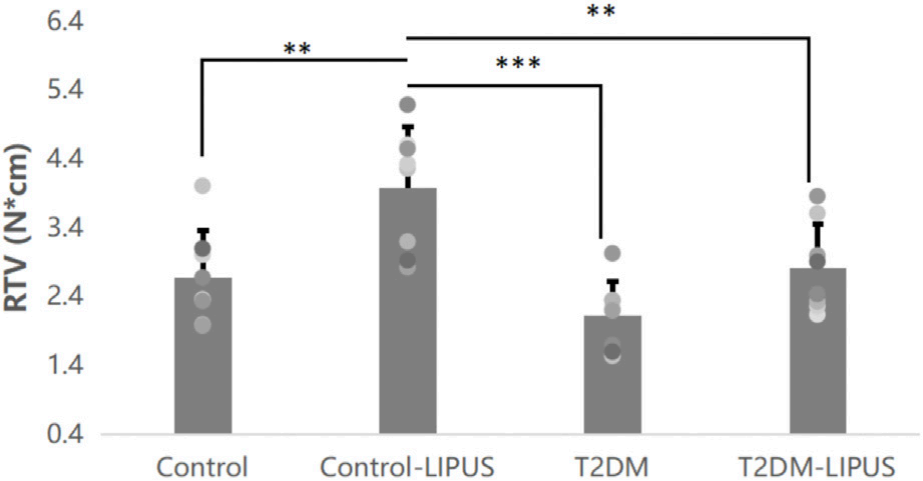
Results of the RTV analysis. LIPUS treatment also significantly increased RTV of healthy rats. There was no difference between Control and T2DM-LIPUS groups. *: *p* < 0.05, **: *p* < 0.01,***:*p* < 0.001. RTV, removal torque value; LIPUS, low-intensity pulsed ultrasound; T2DM, type II diabetes mellitus.

RTV was presented in Figure 7, showing that Control-LIPUS group had significant higher RTV than other three groups.

### 3.5 Characterization of the peri-implant H&E and Masson staining

**FIGURE 8:**
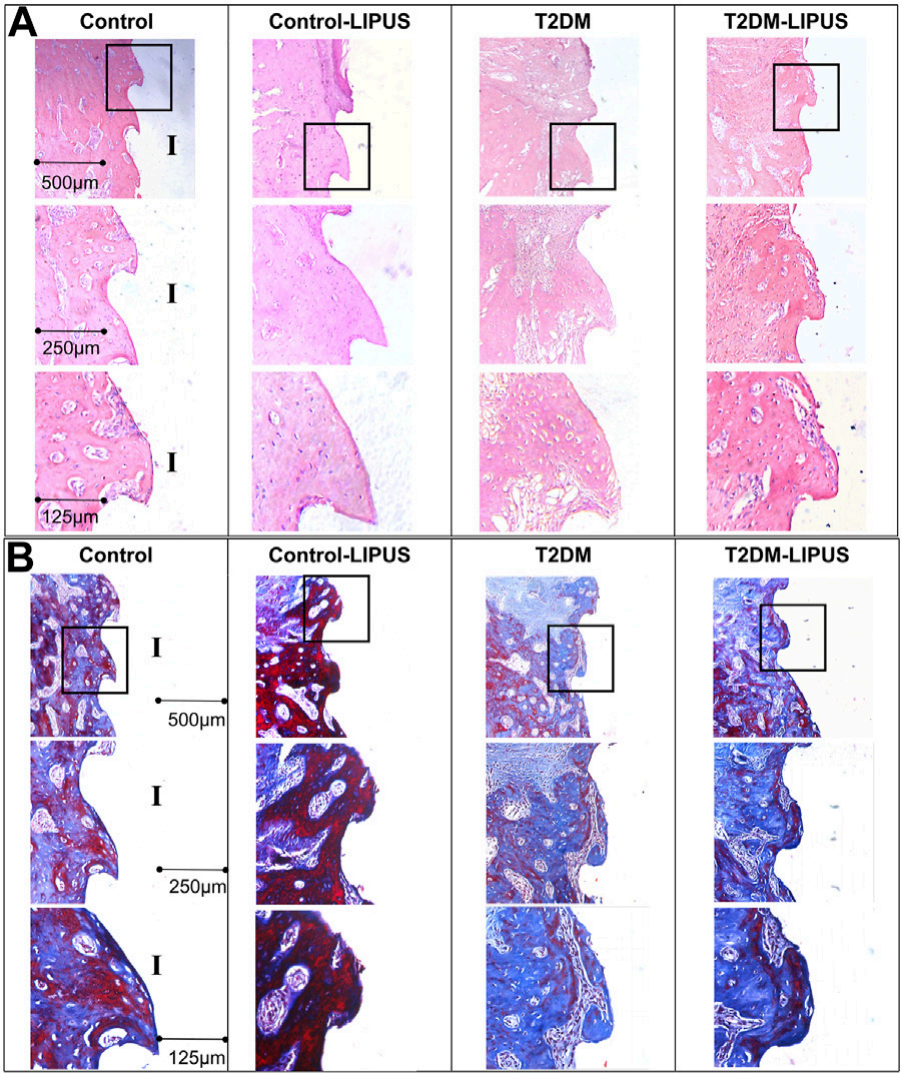
Representative images of H&E and Masson staining in mid-sagittal section of peri-implant bone tissue. **(A)** H&E staining results. **(B)** Masson staining results. The formation of new bone around the implant was more prominent in the LIPUS treated groups, notably the Control-LIPUS group. H&E, hematoxylin and eosin; LIPUS, low-intensity pulsed ultrasound; I, implant area.

The H&E staining and Masson staining were applied to observe the new bone formation around the implant. As shown in Figure 8A, there were more osteoid tissues around implant in Control-LIPUS group than others stained by H&E stain, and LIPUS played a positive role in osteogenic response of peri-implant bone for T2DM rats. In the Masson staining samples, as shown in Figure 8B, the new bone was shown by blue, while the mature bone was shown by red. Mature trabecular bone area was obviously bigger in Control-LIPUS group than others both in T2DM and normal models. What is more, LIPUS treatment improved bone formation in T2DM models. The histologic results were in agreement with the micro-CT evaluation.

## 4 Discussion

Previous studies about the effects of LIPUS on dental implantation have not focused on its effects under T2DM condition. In the present study, the researchers conducted the first evaluation of the effectiveness of LIPUS in stimulating osteogenesis around dental implants in a T2DM rat model. The findings of the study demonstrated a significant and positive impact of LIPUS treatment on promoting osseointegration of dental implants under T2DM conditions.

Though studies have shown that dental implants have a high long-term survival rates and a favorable prognosis (Klokkevold et al., 2007; Chen et al., 2013), it is important to acknowledge that systemic diseases, such as diabetes mellitus, remain significant risk factors that can negatively influence the success rate of dental implantation (Chen et al., 2013). With advancements in implant surface treatment technology, diabetes mellitus with well-managed blood glucose levels is no longer considered an absolute contraindication for implant surgery (Buser et al., 2000). However, several studies have reported that patients with these diseases still pose a potentially high risk of implant failure (Liu et al., 2017; Tang et al., 2021).

T2DM is often associated with various degrees of bone remodeling disorders, which can impede the process of osseointegration during the healing period following implant placement (Liang et al., 2022). Many animal studies have provided confirmation that rats with T2DM exhibit significantly lower osteogenesis level around the implant compared to normal rats (Wang et al., 2010; Hashiguchi et al., 2014; Zhang et al., 2016b), which is also confirmed by the present study’s Micro-CT analysis results, showing that rats in T2DM group had greater cervical marginal bone loss, significant lower BIC ratio, Tb.N, and significant higher Tb.Sp around the implant, compared with those in Control group. Moreover, cellular studies have demonstrated that the high-glucose microenvironment characteristic of T2DM can substantially hinder the osteogenic differentiation and proliferation capacity of BMSCs. It can also decrease the expression of genes related to osteogenesis and slowdown *in vitro* mineralization processes (Gopalakrishnan et al., 2006; Wang et al., 2013; Zhang et al., 2016a).

One clinical study showed statistically significant differences in probing depth (*p* < .00001), marginal bone loss (*p* < .00001), and peri-implant bleeding (*p* < .00001) around the dental implants between the diabetic and non-preexisting disease patients (Jiang et al., 2021). And the incidence of complications with implants was lower in the non-preexisting diseases group (Jiang et al., 2021). Therefore, current clinical researches focus on identifying effective and simple methods to enhance the osseointegration of implants under T2DM condition while also to reduce the duration of the healing process.

LIPUS has been applied in clinical treatment for more than 20 years. LIPUS received approval from the US Food and Drug Administration (FDA) as early as 1994 and 2000 for its use in facilitating the reconstitution of bone nonunion and accelerating the healing process of fresh fractures (Rubin et al., 2001). In recent years, LIPUS has gained widespread recognition as an effective and convenient method for promoting the repairment of bone defects and fracture healing (Harrison and Alt, 2021).

In dentistry, LIPUS has been found effective in promoting periodontal bone defect repairing and bone regeneration after oral maxillofacial surgery (Kim and Hong, 2010; Shiraishi et al., 2011). Besides, clinical studies in orthodontics showed that LIPUS could shorten the whole procedure time of orthodontic treatment (Kaur and El-Bialy, 2020). Some animal experiments have suggested that LIPUS has the potential to influence the osseointegration process and enhance the stability of implants inserted into the femur or tibia of these animals (Ustun et al., 2008; Zhou et al., 2011; Miura et al., 2014; Jiang et al., 2020). Based on the different anatomy, function, and osteogenesis procedure of the long bone from those of alveolar bone (Son et al., 2020), the present study chose maxillae as the implantation site. The implant survival rates in all groups 3 weeks after surgery were 80%, which is similar to the result of a delayed implantation study in rats’ maxillae (He et al., 2022). In the present study, it was found that rats in Control-LIPUS group showed better peri-implant osteogenesis (Tb.Th, BMD, BV/TV, BS/TV, RTV, H&E staining and Masson staining) and lower marginal bone loss than those in Control group, suggesting the positive effect of LIPUS on promoting osteogenesis around the implants.

However, to date, there has been limited clinical research on the application of LIPUS in dental implantation. Only one clinical study conducted by Abdulhameed et al. applied LIPUS to patients with dental implants specifically in the premolar region (Akram et al., 2017). Their findings demonstrated that LIPUS effectively enhanced implant osseointegration and reduced the healing duration. However, as of now, no relevant *in vivo* or clinical studies have reported the effect of LIPUS on osseointegration of implants specifically under diabetic condition and the underlying mechanism. This preliminary *in vivo* study for the first time confirmed that LIPUS promoted osteogenesis around dental implant placed in rats’ maxilla under T2DM condition, as LIPUS treatment significantly increased BIC, Tb.N, Tb.Th, BMD, BV/TV and BS/TV, and significantly decreased marginal bone loss and Tb.Sp of T2DM rats. The present study also showed that LIPUS treatment was able to restore the peri-implant osteogenesis level of the T2DM rats back to the normal level, as confirmed by the same level marginal bone loss, BIC, BS/TV, Tb.Sp, Tb.N, Tb.Th, BMD, BV/TV and RTV of the implants in the LIPUS-treated T2DM rats and the healthy rats.

The potential mechanisms by which LIPUS enhances osteogenesis around dental implants can involve various cellular and molecular processes. Firstly, LIPUS has been shown to stimulate the proliferation and differentiation of osteoblasts through upregulating the expression of key osteogenic genes, including osteocalcin (OCN), runt-related transcription factor 2 (RUNX2), and bone morphogenetic proteins (BMPs) the cells responsible for bone formation (Garg et al., 2017; Liu et al., 2020). This could lead to an increased number of bone-forming cells around dental implants. Besides, LIPUS may enhance the synthesis of extracellular matrix proteins, such as collagen, which are essential for the structural integrity of bone (Kang et al., 2019). LIPUS also has angiogenic effects, promoting the formation of new blood vessels (Kang et al., 2019). Adequate blood supply is crucial for the delivery of nutrients and oxygen to cells involved in bone regeneration, facilitating the overall healing process. Study also showed that LIPUS modulate various signaling pathways involved in bone metabolism, such as the Wnt/β-catenin pathway (Liao et al., 2021). Activation of these pathways can positively influence bone formation and remodeling. Another important function of LIPUS is its anti-inflammatory effects by reducing pro-inflammatory cytokines (Zhang et al., 2020). Lastly, the mechanical forces generated by LIPUS may induce micro-mechanical stress on bone cells, mimicking the physiological loading conditions (Tian et al., 2023). This mechanical stimulation is known to be an important factor in bone remodeling and adaptation. These mechanisms have been observed in various studies, but the exact processes may vary depending on specific experimental conditions. Further research is needed to fully elucidate the intricate mechanisms underlying the beneficial effects of LIPUS on osteogenesis around dental implants.

LIPUS has the advantages of low immunogenicity, low toxicity, high targeting selectivity, noninvasiveness, and repeatability. Based on the reports of clinical studies examining the application of LIPUS, no abnormal reactions or discomfort-related symptoms, such as swelling, redness, or inflammation, have been observed in patients receiving the intervention (Tanaka et al., 2015; Jiang et al., 2019; Tanaka et al., 2020). Moreover, the portable nature of the LIPUS device, which is small and powered by a mobile unit, allows for flexible application without space limitations. Consequently, in the future, LIPUS has the potential to become a safe, effective, and comfortable physical therapy method. It may pave the way for chair-side or at-home treatments for patients with T2DM, ultimately leading to enhanced bone regeneration around the implant, improved implant osseointegration, and even the prevention of marginal bone loss. This, in turn, can significantly enhance the long-term success of dental implant treatment.

Indeed, it is crucial to conduct more prospective cohort studies or randomized controlled trials in the future to further investigate the potential benefits and efficacy of LIPUS in promoting implant osseointegration, especially in patients with T2DM. These studies would provide valuable insights into the optimal treatment protocols, the long-term effects, and the underlying mechanisms of LIPUS therapy.

## 5 Conclusion

This preliminary *in vivo* study showed that LIPUS promoted peri-implant osteogenesis in rats under T2DM condition and found that after LIPUS treatment, the peri-implant osteogenesis level of the T2DM rats increased to the level of normal rats. LIPUS as an effective and safe method has a great potential for T2DM patients in promoting bone regeneration around the implant and achieving higher-quality implant osseointegration. However, in order to validate its clinical function and to investigate its underlying mechanism, further prospective cohort studies or randomized controlled trials are required in the future.

## Data Availability

The original contributions presented in the study are included in the article, further inquiries can be directed to the corresponding authors.
